# The emerging Antarctic amplification

**DOI:** 10.1093/nsr/nwag343

**Published:** 2026-06-04

**Authors:** Qigang Wu, JiKai Tian, Qianyi Yu, Shizuo Liu, Chang Yang, Aixue Hu, Lei Zhang

**Affiliations:** Department of Atmospheric and Oceanic Sciences, Fudan University, China; Key Laboratory of Polar Atmosphere-Ocean-Ice System for Weather and Climate, Ministry of Education, Fudan University, China; Department of Atmospheric and Oceanic Sciences, Fudan University, China; Department of Atmospheric and Oceanic Sciences, Fudan University, China; School of Atmospheric Sciences, Nanjing University, China; Department of Atmospheric and Oceanic Sciences, Fudan University, China; National Center for Atmospheric Research, USA; South China Sea Institute of Oceanology, Chinese Academy of Sciences, China

Although the Antarctic Peninsula was among the most rapidly warming regions during the latter half of the twentieth century, the broader Antarctic continent exhibited weak warming or even cooling, providing little evidence for Antarctic amplification (AnA) [1,2]. Antarctic-mean surface air temperature (SAT) cooled by ∼0.47°C/decade from 1990 to 2014 [3], coincident with a weak multidecadal expansion of Antarctic sea ice extent (ASIE) during 1979–2014. The delayed emergence of AnA has been attributed to several processes, including vigorous Southern Ocean (SO) upwelling and associated ocean heat uptake that is transported away from Antarctica by northward-flowing surface waters [4], enhanced freshwater input from the Antarctic Ice Sheet that cools the SO surface [5], relatively weak lapse-rate and surface albedo feedbacks in SH high latitudes [6], and strong internal variability in regional atmospheric circulation [3,7]. Since 2013/14, Antarctic temperature and sea ice trends have reversed, with a marked decline in ASIE. This decline has been linked to SO subsurface warming, enhanced salinity and upwelling [8–10], the preconditioning effect of a thinned Winter Water layer prior to 2015 [11], and Pacific and Atlantic sub-decadal SST variability [12]. Consistent with this shift, ERA5 shows significant warming south of 60°S of 0.82°C/decade during 2013–25, more than twice the global mean (0.40°C/decade), indicating the emergence of AnA (Fig. 1b).

**Figure 1.**
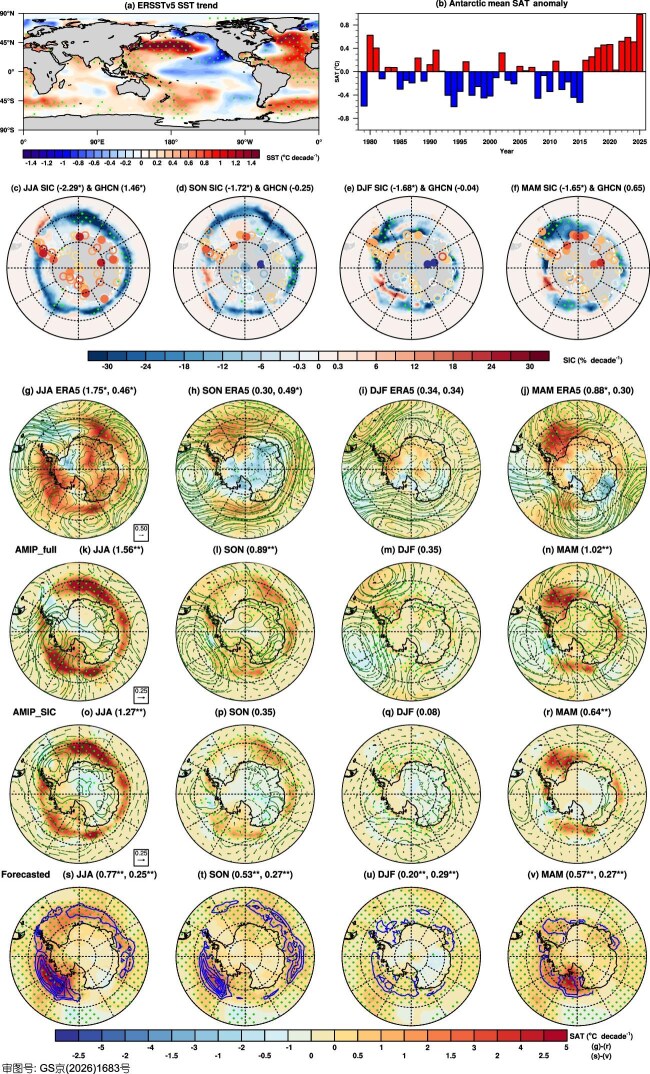
Observed, forced, and predicted Antarctic changes. (a, c–j) Twelve-year observed trends in annual mean SST and seasonal-mean trends in SIC, SAT from GHCN stations and ERA5, SLP (contour interval: 1 hPa/decade), and UV850 from ERA5 during June 2013–May 2025. (b) Antarctic-averaged annual SAT anomalies for 1979–2025. (k–r) Forced 12-year trends in SAT, SLP (contour interval: 0.5 hPa/decade), and UV850 during June 2013–May 2025 derived from the AMIP_Full and AMIP_SIC simulations. (s–v) Predicted 10-year trends in Antarctic SIC (contours; interval: 5%/decade) and SAT from December 2024 to November 2034. Values in parentheses denote seasonal-mean trends in Antarctic sea ice extent (ASIE; 10⁶ km^2^/decade) and Antarctica-averaged GHCN SAT (c–f) and forced Antarctic-mean SAT (k–r), as well as Antarctic- and global-mean SAT from ERA5 (g–j) and the decadal forecasts (s–v), together with their statistical significance. Dotted shading and * indicate trends significant at the 10% level for SST, SIC, and SAT in (a, c–j), whereas dotted shading and ** indicate SAT trends significant at the 5% level in (k–v). Solid blue contours outline negative SIC trends (s–v).

This warming peaks in austral autumn (March–April–May, MAM) and winter (June–July–August, JJA), when it spans much of Antarctica and the surrounding SO, forming a near-circumpolar pattern evident in ERA5 and GHCN (Fig. 1c–j). Seasonal-mean Antarctic (global) SAT trends reach 1.75 (0.46) and 0.88 (0.30)°C/decade in JJA and MAM, yielding amplification factors of ∼3.8 and 2.9 (Text S1). Both East and West Antarctica exhibit strong warming in JJA and MAM (Figs S1 and S2), indicating a transition toward a more spatially coherent, continent-wide signal and a weakening of the long-standing east–west asymmetry (Text S2). In contrast, during austral spring (September–October–November, SON), warming is largely confined to the SO and coastal regions, with weak cooling over the interior, while summer (December–January–February, DJF) shows a weaker and more heterogeneous pattern, including localized warming over the Weddell Sea and Antarctic Peninsula, resulting in only weak Antarctic-mean SAT trends in both seasons. Although the recent ASIE decline is strongly influenced by episodic events—including the abrupt and unprecedented sea ice loss in late 2016 and the year-round decline in 2023—the observed SAT spatial patterns in JJA and MAM (Fig. 1), together with their temporal evolution (Fig. 1b and Figs S1, S2), reveal a persistent, spatially coherent warming signal across Antarctica, indicating a sustained shift toward enhanced Antarctic warming rather than a transient, event-driven response.

We next examine the attribution and predictability of the emerging AnA. Observational evidence indicates that changes in sea ice concentration (SIC), atmospheric circulation, and global SST variability jointly contributed to the observed Antarctic SAT warming (Figs 1b–j). First, SIC declines throughout the year, with the largest reductions near the seasonal ice edge. SIC and SAT trends exhibit strong spatial coherence, with negative SIC anomalies collocated with positive SAT anomalies, consistent with an enhanced ice–albedo feedback [6]. Second, trends in sea level pressure (SLP) and 850-hPa wind vectors (UV850) display pronounced zonal asymmetry across all seasons (Fig 1g–j). In SON and DJF, SLP trends reflect a positive Southern Annular Mode (SAM) and a strengthened Amundsen Sea Low (ASL). In MAM, the circulation trends are dominated by a quasi-stationary asymmetric zonal wave-3 (ZW3) pattern. In JJA, the trends feature a weakened ASL, cyclonic anomalies over the Weddell and Ross Seas, and anticyclonic anomalies extending from the Indian Ocean to the western SO. The spatial pattern of SAT trends is closely tied to the circulation anomalies: regions of strong warming are generally collocated with anomalous northerly flow, while weak cooling corresponds to anomalous southerly flow. Third, the annual SST trend pattern projects onto a negative Interdecadal Pacific Oscillation (IPO) and a positive Atlantic Multidecadal Oscillation (AMO) (Fig. 1a). The raw unfiltered IPO (AMO) index shows a significant downward (upward) trend of −1.76 (0.53)°C/decade from June 2013 to May 2023. Through tropical–polar teleconnections, these modes of decadal SST variability likely contributed to the observed SLP and UV850 trends and thereby to the recent 12-year Antarctic warming [13–15]. By contrast, external radiative forcing accounts for only ∼10%–20% of the observed ASIE decline over the past decade and ∼20% and ∼40% of the Antarctic SAT warming in MAM and JJA, respectively (Fig. S3; Text S3), suggesting a major role of internal atmospheric and oceanic variability in shaping recent Antarctic climate change.

The AMIP_Full experiment, which prescribes observed IPO- and AMO-like SST anomalies together with contemporaneous SIC changes from June 2013 to May 2025 (Text S4), reproduces the major features of the observed circulation response from austral spring to autumn, including a deepened ASL and positive SLP anomalies over the Weddell Sea and the Western South Pacific (Fig. 1k–n). Nevertheless, discrepancies between the simulated and observed SLP trends indicate that additional processes—most notably atmospheric internal variability—also contribute to the observed circulation changes. For example, the observed positive SAM, which is associated with weak Antarctic cooling in SON and DJF (Fig. 1h–i), is not captured by the AMIP_Full simulations. In contrast, the AMIP_SIC experiment produces a substantially weaker circulation response in all seasons (Fig. 1o–r), indicating that the ASIE loss primarily drives strong local SAT warming over the SO through thermodynamic processes rather than large-scale dynamical adjustments. Rapid SIC reductions strongly enhance ocean-to-atmosphere heat fluxes over regions of ice loss, leading to localized tropospheric warming (Figs S4–S6) [16]. The spatial patterns of the turbulent heat flux responses largely mimic those of Antarctic SIC, with the most significant increases over regions of ice loss, particularly in autumn and winter. In addition, SIC loss substantially increases absorbed shortwave solar radiation over the SO from SON through MAM, highlighting the central role of the ice–albedo feedback in Antarctic warming [6]. Consistent with these mechanisms, the AMIP_Full simulation successfully reproduces the observed magnitude and seasonality of Antarctic warming, with the strongest warming along coastal regions (up to 3–5°C/decade in JJA and MAM) extending onto the high-elevation interior plateau. The AMIP_SIC experiment captures the principal SO warming patterns and their seasonal evolution, with pronounced warming over the Antarctic Peninsula and West Antarctica throughout the year, but with only limited cooling over East Antarctica. Quantitatively, ASIE decline accounts for approximately 78%, 45%, 14%, and 50% of the total AMIP_Full Antarctic warming in JJA, SON, DJF, and MAM, respectively.

Decadal forecasts, initialized with observed oceanic and atmospheric conditions in November 2024 and driven by projected external forcing (Text S1), predict widespread reductions in Antarctic SIC and pronounced SAT warming during 2025–34 (Fig. 1s–v). Although the projected SIC decline and Antarctic warming rates are substantially weaker than those observed over the past decade, AnA is nevertheless expected to persist, with Antarctic-to-global warming ratios of ∼2–3 in MAM, JJA, and SON, consistent with the forecasted SIC reductions. While there is substantial inter-model and intra-model spread in the projections (Figs S7 and S8), the sign of the response is robust across models, indicating that the persistence of AnA is more certain than its magnitude and regional expression. This predictability arises from the combined effects of SO subsurface heat memory, large-scale atmospheric circulation constraints, and increasing external radiative forcing (Text S5, Figs S9–S12).

Recent studies have documented the rapid decline of ASIE over the past decade and the associated abrupt changes in the Antarctic environment [8–12,17]. Here, we provide robust evidence of widespread Antarctic surface warming and the emergence of AnA, which is most pronounced during austral autumn and winter. This warming is primarily driven by ASIE loss, with an additional contribution from decadal-scale SST variability. The vertical structure of AnA exhibits a pronounced enhancement in the lower and middle troposphere during JJA, with amplification factors exceeding 2.0 (Text S6 and Figs S13 and S14), closely resembling the well-documented vertical profile of Arctic amplification in boreal winter. The ASIE decline itself is closely linked to the negative IPO-like Pacific decadal SST trend (Fig. 1a) [12], indicating that internal atmospheric and ocean variability has played a dominant role in recent Antarctic warming and the emergence of AnA. Persistent, year-round Antarctic warming is expected to accelerate ice-shelf mass loss and further destabilize the Antarctic cryosphere [18]. The emergence of AnA thus represents a key manifestation of recent abrupt changes in the Antarctic climate system, with far-reaching implications for ice-shelf stability, global sea-level rise, climate variability, marine heatwaves, and SO ecosystems. Together with anthropogenic forcing, continued ASIE decline and sustained SO heat release provide a physically consistent basis for the persistence of Antarctic warming—and hence AnA—over the coming decade. Under a 2°C global warming scenario, projected to be reached in the 2040s under SSP3-7.0, climate models robustly simulate continent-wide Antarctic warming and a clear AnA signal [19]. Nevertheless, the rate, seasonality, and regional expression of future AnA will depend on the combined influences of internal variability, Antarctic Ice Sheet meltwater forcing, and external radiative forcing (Text S7).

## Supplementary Material

nwag343_Supplemental_File
