# Supplementary material for: The emerging Antarctic amplification
Source: Natl Sci Rev. 2026 Jun 4;13(12):nwag343. doi: 10.1093/nsr/nwag343 (PMC13325488; doi:10.1093/nsr/nwag343)
Supplement: nwag343_Supplemental_File [file nwag343_supplemental_file.docx]

**Supplementary Information**

**The Emerging Antarctic Amplification**

Qigang Wu^1,2^*, JiKai Tian^1^, Qianyi Yu^1^, Shizuo Liu^3^, Chang Yang^1^, Aixue Hu^4^, and Lei Zhang^5^

^1^Department of Atmospheric and Oceanic Sciences, Fudan University, Shanghai, 200438, China

^2^Key Laboratory of Polar Atmosphere-Ocean-Ice System for Weather and Climate, Ministry of Education, Fudan University, Shanghai, 200438, China

^3^School of Atmospheric Sciences, Nanjing University, Nanjing, 210023, China

^4^National Center for Atmospheric Research, Boulder, 80307, USA.

^5^South China Sea Institute of Oceanology, Chinese Academy of Sciences, Guangzhou, 510301, China

Corresponding author: Dr. Qigang Wu, Email: [qigangwu@fudan.edu.cn](mailto:qigangwu@fudan.edu.cn)

**This file includes:**

Supplementary text S1-S7

Supplementary Figure S1-S14

SI References

**S1:** **Observational and modeling datasets and analysis**

We use multiple observational and reanalysis datasets. Monthly sea ice concentration (SIC) is obtained from a newly updated homogeneous, high-resolution dataset derived from two established passive-microwave algorithms: the NASA Team (NT) and NASA Bootstrap (BT) algorithms [18-19]. Atmospheric fields are taken from the ERA5 reanalysis [20]. Antarctic surface air temperature (SAT) station observations are obtained from the Global Historical Climatology Network (GHCN) [21], and sea surface temperature (SST) data are taken from the Extended Reconstructed Sea Surface Temperature version 5 (ERSSTv5) dataset [22]. In the GHCN dataset, a total of 69 stations are available during June 2013–May 2025, including 39 stations in East Antarctica and 30 in West Antarctica. Ocean subsurface temperature data are derived from the extension of the gridded Argo data product [23]. The Interdecadal Pacific Oscillation (IPO) and Atlantic Multidecadal Oscillation (AMO) indices are obtained from the Earth System Research Laboratory (ESRL) of the National Oceanic and Atmospheric Administration (NOAA) and are calculated using ERSSTv5 SST fields. The IPO index is defined as the Tripole Index (TPI), calculated as the difference between the SST anomalies averaged over the central equatorial Pacific and the mean SST anomalies over the northwest and southwest Pacific. The AMO index is calculated as the area-weighted mean SST anomaly over the North Atlantic (0–70° N).

Linear trends are calculated for annual and seasonal mean anomalies of Antarctic SIC and ASIE, ERSSTv5 SST, ERA5 atmospheric fields, and zonally averaged subsurface temperature over 50-65°S (denoted as TEMP_SO) at 0-200 m depth over the 12-year period from June 2013 to May 2025. Trends in SAT averaged over 60–90° S (Antarctic) are compared with global mean SAT trends to assess the emergence of AnA. An Antarctic-to-global warming ratio of 2 or greater is considered indicative of a robust AnA signal.

To estimate climate responses to radiative forcing, we analyze multi-model ensemble mean 12-year trends over June 2013 to May 2025 from CMIP6 models [24] that have at least three ensembles of historical and SSP3-7.0 future radiative forcing scenarios, and a realistic climatology of sea ice concentration (SIC) for each scenario simulation to reduce the impacts of large internal variability on the 12-year trends in SIC, SAT, subsurface temperature, zonal mean zonal temperature ([T]), zonal mean wind ([U]) and zonal mean geopotential height ([Z]). To match observations, simulations from the historical and SSP3-7.0 scenario experiments are concatenated over June 2013 to May 2025. Very similar forced trends are obtained when using SSP2-4.5 instead of SSP3-7.0.

We further analyze decadal forecasts of SIC and SAT from 76 ensemble members across four climate models: BCCR (30 members), CMCC (20), MOHC (10) and MPI/DWD (16), obtained from the WMO Lead Centre for Annual-to-Decadal Climate Prediction [25]. The forecasts are initialized in November 2024 and extend through December 2034, with prescribed external forcing following the SSP2-4.5 scenario [24]. Ten-year linear trends of SIC and SAT are calculated for the period December 2024 to November 2034. Because forecast SIC anomalies are provided relative to the 1991–2020 climatology, only Antarctic sea ice area (ASIA), rather than ASIE, trends are analyzed in the forecasts. The projected ASIA trends are −1.06, −1.13, −0.85 and −0.49 × 10⁶ km²/decade in JJA, SON, DJF and MAM, respectively, which are weaker than the corresponding observed ASIA trends of −2.02, −1.55, −1.38 and −1.36 × 10⁶ km²/decade from June 2013 to May 2025. Note that none of the above four decadal prediction models used in this study explicitly includes glacial meltwater forcing from the Antarctic ice sheet, which may influence upper-ocean stratification and sea ice evolution.

For significance testing, we calculate the standard deviation (σ) of 12-year linear trends at each grid point or over specified regions using all available ensemble members from the CMIP6 simulations. A two-sided local significance test is applied to assess whether an observed trend (|trend| ≥ 2.0σ) or the ensemble-mean trend (|trend|≥2.0σ/$\sqrt{\text{Ne}}$) differs significantly from zero at the 5% significance level. The same approach is applied to assess the statistical significance of the forecasted 10-year trends in SIC, SAT, and ASIA.

**S2. Consistency of SAT trends between ERA5 and GHCN**

To assess the robustness of the observed Antarctic warming, we compare SAT trends from ERA5 and GHCN over East and West Antarctica (Figs. S1–S2), in conjunction with the spatial patterns shown in Fig. 1. Both datasets exhibit consistent seasonal and regional characteristics.

In JJA, pronounced and statistically significant warming is evident in both East and West Antarctica (ERA5: 2.15 and 1.39°C/decade; GHCN: 1.68 and 1.34 °C/decade), indicating a transition toward a more spatially coherent, continent-wide warming signal, consistent with the near-circumpolar pattern in Fig. 1. In MAM, warming remains strong in West Antarctica (0.79°C/decade in GHCN and 1.09°C/decade in ERA5), while East Antarctica exhibits weaker and less statistically significant trends (0.44°C/decade in GHCN and 0.58°C/decade in ERA5), suggesting that some degree of east–west asymmetry persists during this transitional stage. In contrast, during austral summer and spring (DJF and SON), trends are weaker and more spatially heterogeneous, with insignificant warming in West Antarctica and weak cooling in East Antarctica in both datasets, consistent with the more localized and less coherent patterns in Fig. 1.

Overall, the agreement between ERA5 and GHCN in both the seasonal evolution and spatial structure of SAT trends supports the robustness of the emerging AnA signal and indicates a marked reduction of the historical east–west asymmetry since the mid-2010s, particularly during JJA and MAM.

**S3. Contributions of external radiative forcing to Antarctic sea ice reduction and SAT warming**

CMIP6 multi-model ensemble simulations are used to estimate the contribution of external radiative forcing to ASIE and SAT trends from June 2013 to May 2025 (Supplementary Text S1; Fig. S3). The forced response exhibits widespread but relatively weak Antarctic SIC decline and SAT warming (Fig. S3), in contrast to the much stronger and more spatially coherent trends in observations (Fig. 1). Quantitatively, external forcing accounts for only ~10–20% of the strong ASIE decline in four seasons in observations, and ~20% (JJA) and ~40% (MAM) of the observed ERA5 Antarctic SAT warming during the seasons when AnA is most pronounced.

Furthermore, the global-mean SAT trends in Fig. S3 indicate that Antarctic warming under external forcing does not exceed the global mean, implying that AnA does not emerge in CMIP6 forced simulations alone. This discrepancy between the forced response and observations underscores the dominant role of internal atmospheric and oceanic variability—particularly coupled ocean–atmosphere processes—in shaping the recent Antarctic sea ice decline and amplified warming on decadal (∼12-year) timescales.

**S4: AMIP simulations**

The relatively small contribution of external radiative forcing to the observed Antarctic-mean surface warming and ASIE decline during 2013–2023 highlights the dominant role of natural internal variability in recent Antarctic climate change. Such variability includes decadal SST anomalies associated with the negative IPO and positive AMO, as well as atmospheric circulation changes (Fig. 1). The raw, unfiltered IPO (AMO) index exhibits a significant negative (positive) linear trend of −1.76 (0.53) °C/decade over the period June 2013 to May 2025. In addition, a positive phase of the SAM contributed to cold temperature trends over the Antarctic interior (Fig. 1h–i) [26], while enhancing warming over parts of the SO through circulation-driven heat advection. Consistent with this, Antarctic-mean SAT trends over 50–90°S in SON and DJF are weak and not statistically significant, and no clear AnA signal emerges in these two seasons.

We conduct 20-member ensemble simulations using Atmospheric Model Intercomparison Project (AMIP)-type uncoupled atmospheric experiments forced with observed, monthly varying SSTs and sea ice concentrations (SICs), while greenhouse gas concentrations are fixed at 2010 levels. These simulations span January 2013 to July 2025 and are referred to as AMIP_Full. Ensemble-mean fields from the AMIP_Full simulations provide an estimate of the forced atmospheric response to the observed decadal SST variability—primarily associated with the negative IPO and positive AMO (Fig. 1a)—together with Antarctic SIC loss over this period. Decadal variability in Pacific and Atlantic SSTs has been shown to influence Antarctic atmospheric circulation on decadal time scales [13–15]. Using coupled model experiments, Wu et al. (ref. 12) further demonstrated that the Pacific sub-decadal SST trend during June 2013 to May 2023 (their Fig. 2a), which is similar in pattern but stronger in magnitude than that shown in Fig. 1a, predominantly drove contemporaneous changes in Antarctic atmospheric circulation and contributed to SO subsurface warming and ASIE decline. Discrepancies between the forced SLP trends in the AMIP_Full simulations and the corresponding observed trends suggest that additional processes, including atmospheric internal variability such as SAM (Figs. 1h–i), also contribute to the observed Antarctic circulation changes.

A second set of 20-member ensemble simulations is performed with SIC identical to that in AMIP_Full but with SSTs replaced by a repeating climatological seasonal cycle for 2013–2025. These simulations, referred to as AMIP_SIC, are designed to isolate the atmospheric response to Antarctic sea ice changes. All simulations are conducted using the Community Atmosphere Model version 5.1 (CAM5.1) [27], run at a horizontal resolution of 0.94° latitude × 1.25° longitude with 18 vertical levels. In regions where sea ice cover varies, SSTs are allowed to adjust accordingly [28]. Although AMIP_SIC may underestimate the long-term atmospheric response to Antarctic sea ice loss [16], it is siutable for quantifying the atmospheric response to the observed ASIE decline over the study period.

For significance testing, we calculate the standard deviation (σ) of 12-year linear trends at each grid point or over specified regions using the 20 ensemble members from the AMIP_Full and AMIP_SIC experiments, with ensemble members (Nₑ = 20) assumed to be independent.

**S5. Inter-model and intra-model spread, sources of decadal prediction skill**

Figures S7–S8 illustrate the inter-model and intra-model spread of decadal (December 2024–November 2034) trends in ASIA and SAT. Substantial spread is evident both across models and among ensemble members within each model, highlighting the strong influence of internal variability and differences in model physics on decadal Antarctic climate projections. The magnitude of the spread varies by model and season, with particularly large intra-model dispersion in JJA and SON, indicating considerable uncertainty in the amplitude and regional expression of the projected changes. Despite this spread, the ensemble-mean signals are broadly consistent across all four prediction models. Most ensemble members simulate declining ASIA and increasing SAT, and the ensemble means consistently indicate ASIA loss and warming across seasons. This cross-model agreement suggests that the projected continuation of Antarctic sea ice decline and warming is robust in sign, although uncertain in magnitude and regional details.

This robustness likely arises from several physically grounded sources of predictability.

First, observations show that recent Antarctic warming has been accompanied by a sustained increase in SO subsurface heat content since the early 2000s, providing a persistent oceanic heat reservoir that influences sea ice through enhanced ocean-to-ice heat flux from below. Zonally averaged subsurface temperature over 50-65°S (TEMP_SO) anomalies derived from Argo observations reveal pronounced subsurface warming since 2004 (Fig. S9). The annual-mean TEMP_SO averaged over the 0–200 m layer exhibits a significant warming trend of ~0.12 °C/decade during 2004–2024, strengthening to ~0.18 °C/decade over 2014–2024. Both trends are statistically significant at the 5% level. The recent acceleration of upper-ocean subsurface warming indicates an intensification of the oceanic heat reservoir, providing sustained preconditioning for the ongoing decline of Antarctic sea ice. Decadal hindcasts further indicate that approximately half of the observed ASIE difference between high-ice (2012–2015) and low-ice (2017–2020) periods can be attributed to subsurface ocean warming [8].

Second, although SST outputs are not available from the decadal forecast archive used here, the simulated large-scale atmospheric circulation and SAT responses provide indirect but physically meaningful constraints on the underlying SST forcing. The forecasts consistently exhibit a deepened ASL and widespread warming over the SO and Antarctica (Figs. S10–11), consistent with atmospheric teleconnections driven by tropical variability. Although a positive IPO phase is generally unfavorable for Antarctic sea ice decline, our results suggest that other forcings—particularly tropical Atlantic warming—can play a compensating role. Tropical Atlantic SST anomalies can excite stationary Rossby wave trains that propagate into SH high latitudes, inducing ASL deepening and associated circulation changes that promote sea ice loss [13], consistent with the projected SLP patterns (Fig. S11).

Third, long-term SO warming since the 1950s has been robustly attributed to increasing anthropogenic greenhouse gas concentrations [29]. To further assess the role of external forcing in recent decades, we analyze forced trends of the 0-200 m layer mean TEMP_SO in CMIP6 simulations (Fig. S12). The simulated trends in annual TEMP_SO at 0–200m depth during 2004–2024 and 2014–2024 are approximately 0.11 °C/decade, comparable to the observed trend over 2004–2024 (0.12 °C/decade), but smaller than the stronger warming observed during 2014–2024 (0.18 °C/decade) in Argo. Coupled experiments further suggest that Pacific sub-decadal SST variability has contributed substantially to the enhanced subsurface warming in the SO and associated Antarctic sea ice decline during 2013–2023 [12]. These results indicate that anthropogenic radiative forcing provides a key background warming trend in the SO, while internal variability—particularly Pacific SST-driven circulation changes—amplifies the observed warming on decadal timescales.

CMIP6 simulations further indicate that anthropogenic forcing will continue to enhance SO subsurface warming in the coming decades. The projected trends in the 0-200 m layer mean TEMP_SO are approximately 0.12 °C/decade for 2025–2034 and 0.27 °C/decade for 2025–2044 (Fig. S12). This sustained subsurface warming is expected to further precondition the upper ocean, favoring continued Antarctic sea ice decline and supporting the persistence of AnA over the coming decades.

In summary, these results suggest that decadal predictability in the Antarctic does not arise solely from externally forced trends, but from the combined effects of SO heat content memory, large-scale atmospheric circulation constraints, and background radiative forcing. Together, these processes provide a physically consistent basis for near-term predictability, with greater confidence in the direction of change than in its magnitude.

S**6: Vertical structure of AnA**

Both observations and simulations consistently indicate that AnA occurs in MAM and JJA in the 12-year period. The vertical structure of Antarctic temperature trends reveals warming throughout the troposphere, with the strongest warming in JJA and a pronounced near-surface signal, particularly below 850 hPa (Figs. S13-S14). In MAM, the warming is weak but statistically significant, and it is confined to altitudes below 650 hPa. Profiles of annual and seasonal AnA factors, calculated as the ratio of the Antarctic mean [T] trend to that of the global mean [T] in each level, indicate that the AnA factor is greater than 2.0 in the low and middle troposphere in JJA (Fig. S14d), but only in the lower troposphere in MAM. During the austral winter, weak midlatitude cooling is observed in the mid-troposphere, significantly reducing the equator-to-pole temperature gradient. Consistent with hydrostatic balance, zonal-mean geopotential heights ([Z]) increase in response to tropospheric warming, with the largest increases occurring in the upper troposphere. This height response is associated with a weakening of the tropospheric eddy-driven jet and projects onto the negative phase of the SAM (Figs. S14b–c).

The zonal-mean temperature response to remote SST and Antarctic sea ice loss differs across experiments (Figs. S14f–g). In the AMIP_Full, warming extends through the troposphere from 40°–80°S, peaking over the high southern latitudes in the lower troposphere, while polar cooling occurs poleward of 80°S. In AMIP_SIC, warming is restricted to the lower troposphere (to ~500 hPa) over 55°–70°S, coinciding with sea ice loss, while cooling dominates over the Antarctic interior. In JJA, the general pattern of the zonal-mean response to radiative forcing in CMIP6 shows widespread and deep tropospheric warming in middle and high latitudes, with the strongest (weakest) warming near the surface across the Antarctic (midlatitude Southern Ocean) (Fig. S14e). The observed deep tropospheric Antarctic warming and associated reduction in mid-latitude temperature gradients is largely consistent with contributions from global decadal SST variations and radiative forcing (Fig. S14h).

**S7. Uncertainties in projected AnA**

ASIE loss has contributed substantially to the emergence of AnA. Decadal hindcasts suggest that approximately half of the observed ASIE contrast between high-ice (2012–2015) and low-ice (2017–2020) periods can be attributed to subsurface ocean warming [8], with the remainder arising from coupled atmosphere–ocean variability. Consistently, coupled pacemaker experiments indicate that sub-decadal SST trends—primarily in the Pacific, with additional contributions from tropical Atlantic warming—account for ~40% of the observed ASIE decline during June 2013–May 2023 [12]. In addition, declining sea ice may in turn intensify storm activity, particularly in winter [30]. Observational and reanalysis evidence suggests an increase in storminess in recent decades [31], which can enhance upper-ocean vertical mixing and upward heat transport in the Southern Ocean. These storm-related processes likely act as a local positive feedback, amplifying sea ice loss by strengthening ocean–atmosphere coupling and facilitating the release of subsurface heat. Despite these advances, a comprehensive understanding of the mechanisms governing Antarctic sea ice variability and associated Southern Ocean processes—such as subsurface temperature and salinity changes—remains limited, owing to sparse observations and persistent model deficiencies. These mechanistic uncertainties in recent Antarctic sea ice decline propagate into uncertainties in the evolution and predictability of AnA, as discussed below.

Substantial uncertainties therefore remain in the projected rate and regional expression of future Antarctic sea ice change and AnA. Previous studies indicate that increased meltwater input can substantially alter upper-ocean stratification and, consequently, sea ice evolution [5], yet future glacial meltwater input remains highly uncertain [32]. Notably, none of the four decadal prediction models, nor the CMIP6 models used here for Antarctic SAT and TEMP_SO analyses, includes glacial meltwater forcing from the Antarctic Ice Sheet. In addition, Antarctic ice–ocean–atmosphere coupled climate models continue to struggle to reproduce the observed variability of Antarctic sea ice [33], highlighting persistent structural deficiencies. Internal atmospheric and oceanic decadal variability, together with the absence of meltwater forcing and inherent model limitations, therefore represent major sources of uncertainty in projections of Antarctic sea ice change and AnA. Thus, although a robust AnA is projected for 2025–2034, its persistence on longer timescales will depend on the interplay among internal variability, Antarctic Ice Sheet meltwater forcing, and external radiative forcing.

Nevertheless, sustained subsurface heat accumulation in the Southern Ocean (Figs. S9 and S12) provides a physically consistent mechanism for continued upper-ocean preconditioning, favoring ongoing sea ice decline and supporting the persistence of Antarctic warming on decadal timescales despite these uncertainties.

**Data availability**

All observational and decadal forecast datasets used in this study are publicly available. Antarctic sea ice concentration data are obtained from the National Snow and Ice Data Center (NSIDC) (https://nsidc.org). ERA5 reanalysis data are available from the European Centre for Medium-Range Weather Forecasts (ECMWF) (https://www.ecmwf.int). Sea surface temperature data from ERSSTv5 data are available from the NOAA Earth System Research Laboratory (ESRL) Physical Sciences Laboratory (https://psl.noaa.gov). Argo ocean temperature data is from <https://sio-argo.ucsd.edu/RG_Climatology.html>. Decadal forecasts of SIC and SAT from the four models analyzed here are available from the WMO Lead Centre for Annual-to-Decadal Climate Prediction (http://gws-access.jasmin.ac.uk/public/wmo_ntcp/). Output from the AMIP simulations generated in this study is available at https://doi.org/10.5281/zenodo.20682797.

**References**

18. Cavalieri DJ, Gloersen P, Campbell WJ. *J Geophys Res* 1984*;* **89**: 5355–5369.

19. Comiso JC, *et al.* *J Clim* **30**, 2251–2267 (2017).

20. Hersbach H, Bell B, Berrisford P, *et al.* *Q J R Meteorol Soc* 2020; **146**:1999–2049.

21. Menne MJ, Durre I, Vose RS, et al. *J Atmos Ocean Technol* 2012; **29**: 897–910.

22. Huang B, Thorne PW, Banzon VF, *et al.* *J Clim* 2017; **30**: 8179–8205.

23. Roemmich D, Gilson J. Prog Oceanogr 2009; 82: 81–100.

24. Eyring V, *et al.* *Geosci Model Dev* 2016; **9**: 1937–1958.

25. Hermanson L, *et al.* *Bull Am Meteorol Soc* 2022; **103**: 1117–1129.

26. Thompson DWJ, Solomon S. *Science* 2002; **296**: 895–899.

27. Neale RB, *et al.* *NCAR Tech Note* NCAR/TN-486+STR (Natl Cent Atmos Res, 2012).

28. Screen JA, Simmonds I, Deser C, Tomas R. *J Clim* 2013; **26**:1230–1248.

29. Swart NC, Gille ST, Fyfe JC, Gillett NP. *Nat Geosci* 2018; **11**:836–841.

30. Josey SA, Meijers AJ, Blaker AT, et al. *Nature* 2024; **636**:635-639.

31. Lin X, Zhai X, Wang Z, Munday D *J Clim* 2018; **31**:3557–3573.

32. Seroussi H, Nowicki S, Payne AJ, et al. *Cryosphere* 2020;14:3033–3070.

33. Turner J, Bracegirdle TJ, Phillips T, et al. *J Clim* 2013; 26:1473-1484.


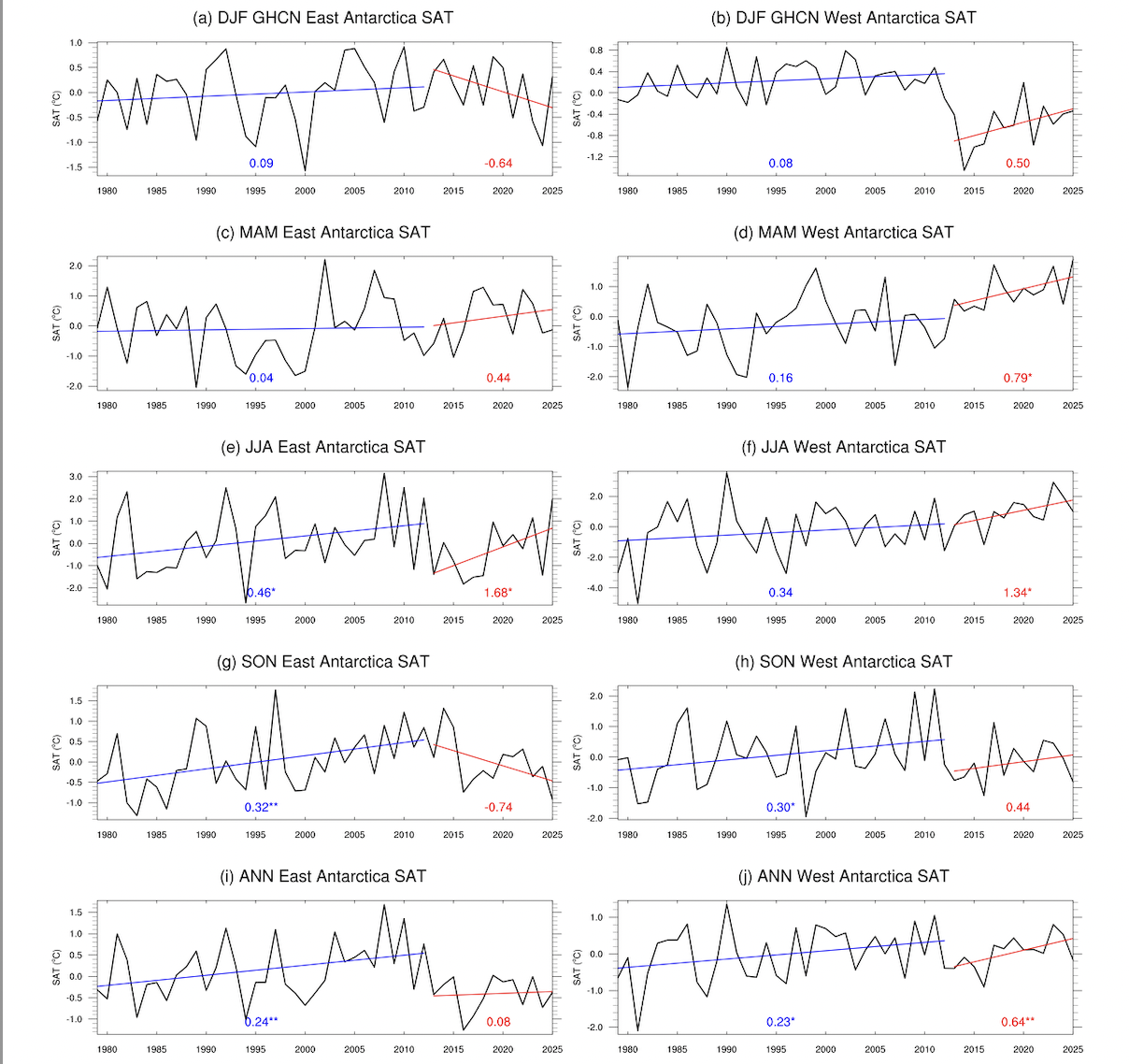


**Fig. S1.** Time series of seasonal and annual mean surface air temperature (SAT) anomalies over Antarctica for June 1979–May 2025 from the GHCN dataset, averaged over East Antarctica (left column) and West Antarctica (right column). Linear trends for June 1979–May 2013 (blue) and June 2013–May 2025 (red) are shown, with corresponding trend values (°C/decade) indicated in each panel. Asterisks denote statistical significance, with ** and * indicating significance at the 5% and 10% levels, respectively. The number of available stations increases from 30 (13 in East Antarctica and 17 in West Antarctica) prior to 2013 to 69 (39 in East Antarctica and 30 in West Antarctica) after 2013. The pre-2013 period is provided as a baseline for context, whereas the post-2013 evolution is the primary focus of this analysis. Notably, the 2013–2025 SAT time series and corresponding trends over both East and West Antarctica, when calculated using the same set of 30 stations as in 1979–2012, are highly consistent with those derived from all 69 stations in the later period, in both magnitude and seasonal structure, demonstrating the robustness of the results.

.


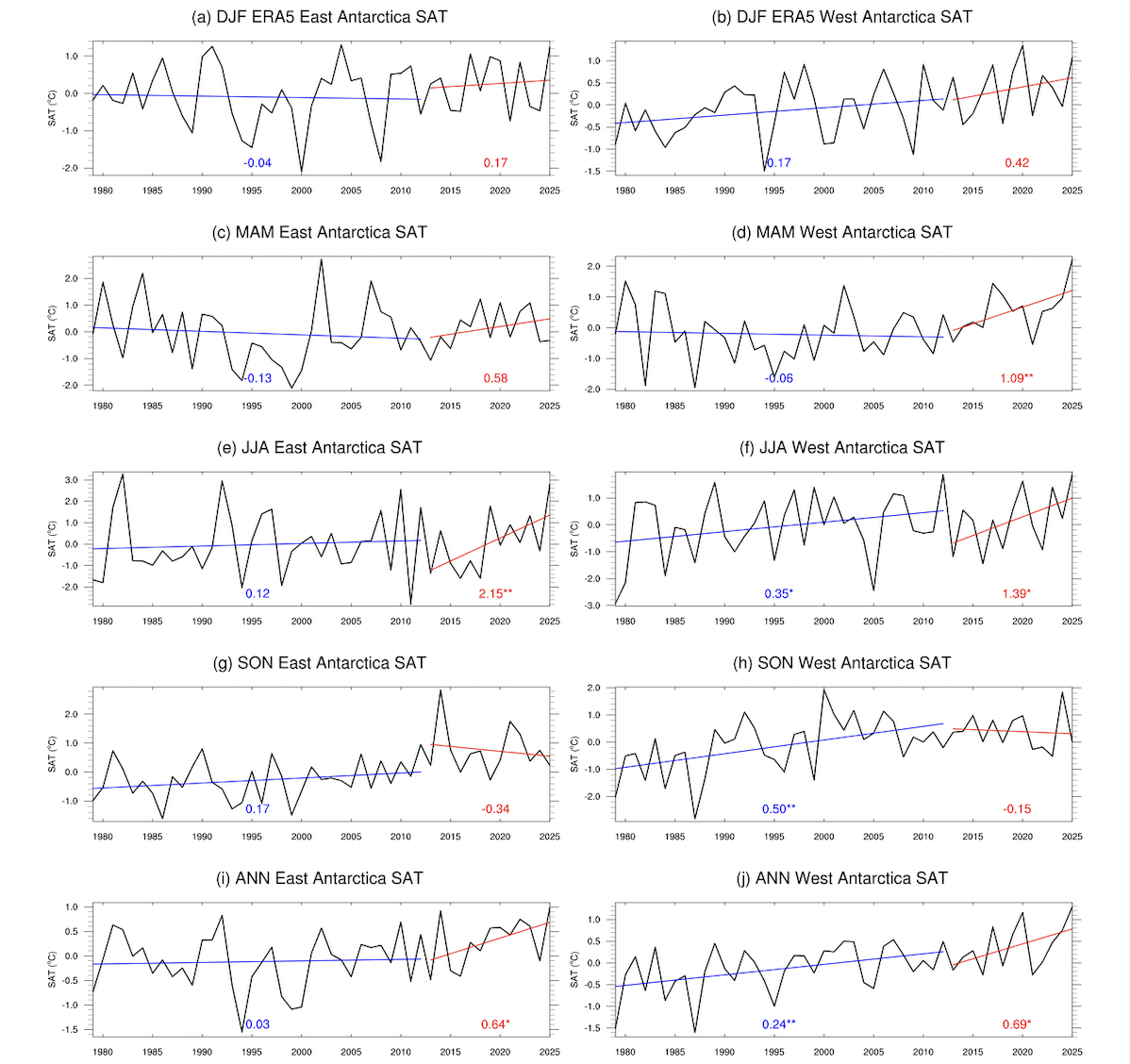


**Fig. S2.** Same as Fig. S1, but for SAT from the ERA5 reanalysis.


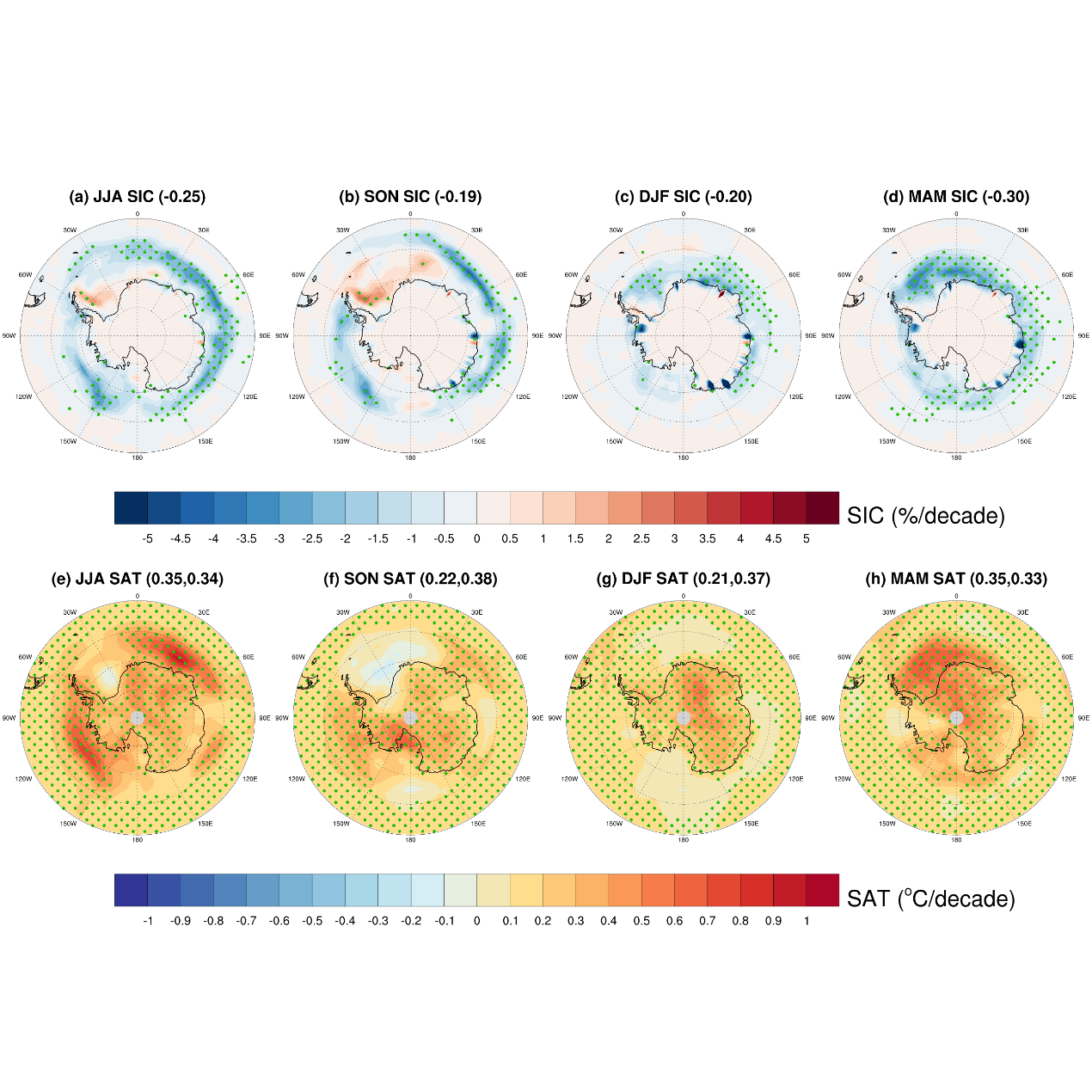


**Fig. S3.** Contributions of external radiative forcing to recent Antarctic climate trends. Ensemble-mean 12-year linear trends (June 2013–May 2025) of seasonal Antarctic (a–d) SIC (%/decade) and (e–h) SAT (°C/decade). Stippling denotes regions where trends are significant at the 5% level. Numbers in parentheses indicate seasonal-mean trends of ASIE (10⁶ km²/decade) and area-averaged SAT (Antarctic: 60–90°S; global), respectively; all values are statistically significant at the 5% level.


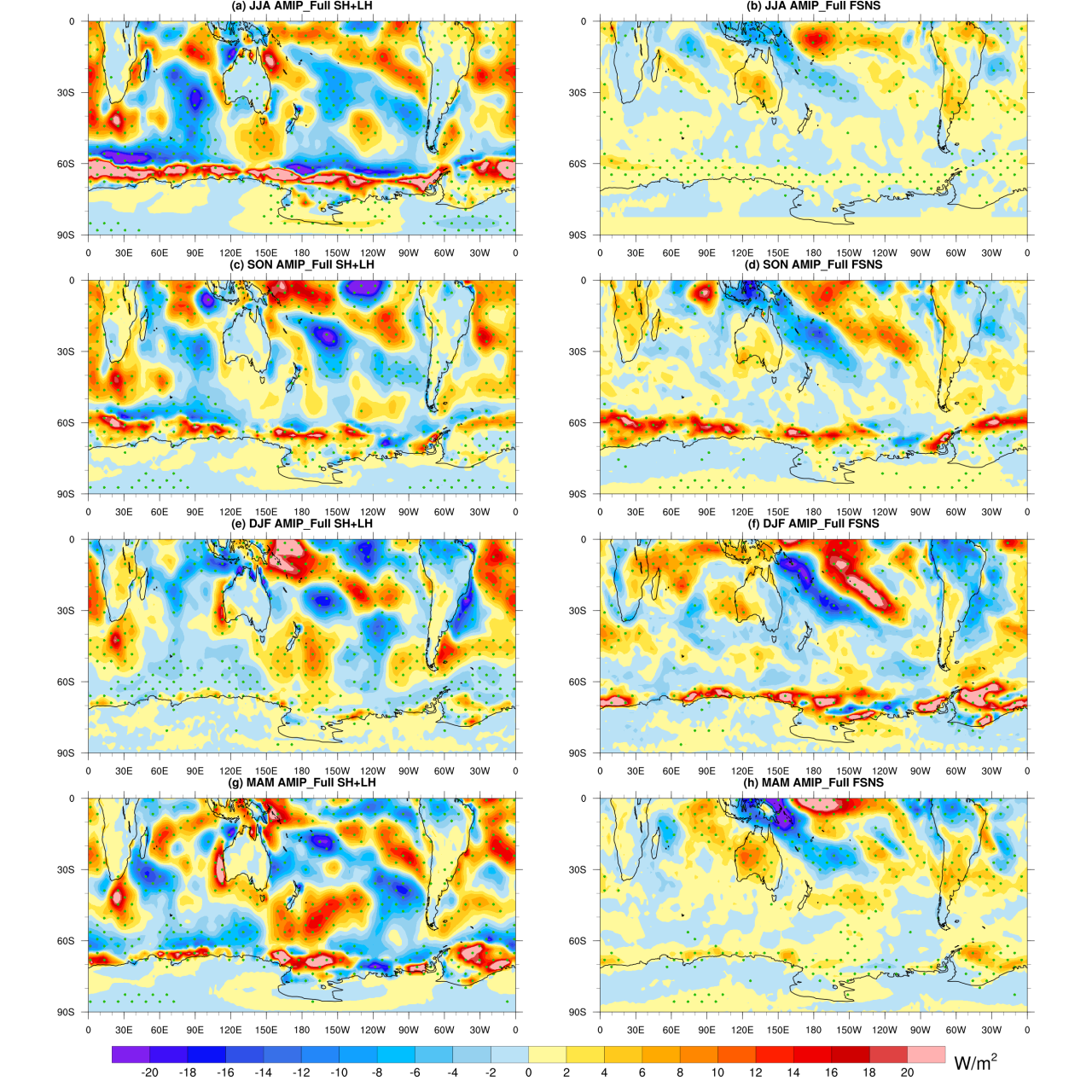


**Fig. S4.** **Forced trends in surface heat flux in the AMIP_Full experiment.** 12-year trends in (i) net surface latent heat flux (LH) plus sensible heat flux (SH), and (ii) net short-wave radiation (FSNS) in the AMIP_Full experiment. The LH and SH fluxes are defined as positive in the upward direction, so red denotes areas where the heat flux into the atmosphere is higher in the experiments. Dotted areas denote trend values significant at the 5% significance level.


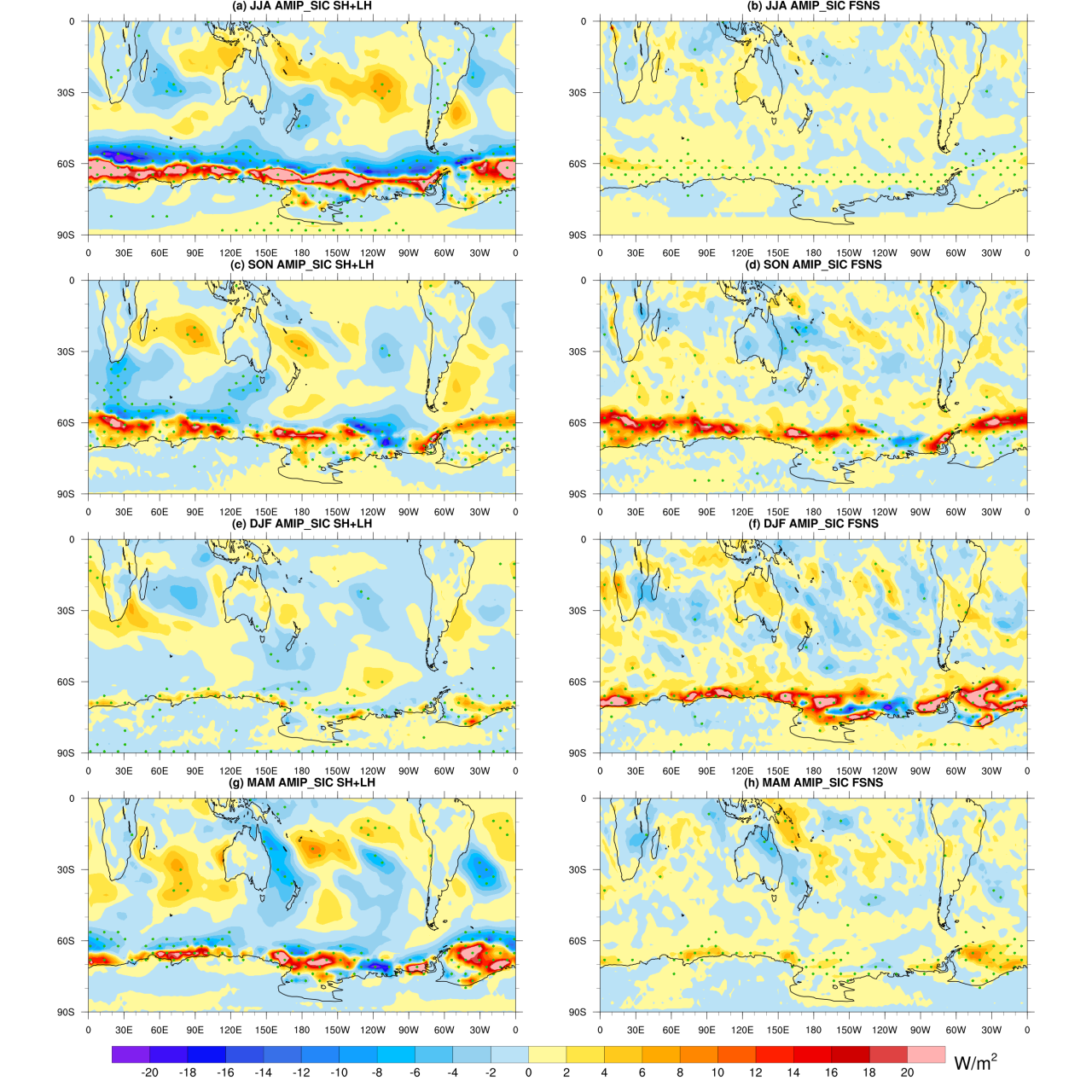


**Fig. S5.** **Forced trends in surface heat flux in the AMIP_SIC experiment.** Same as Fig. S4 except for the AMIP_SIC experiment.


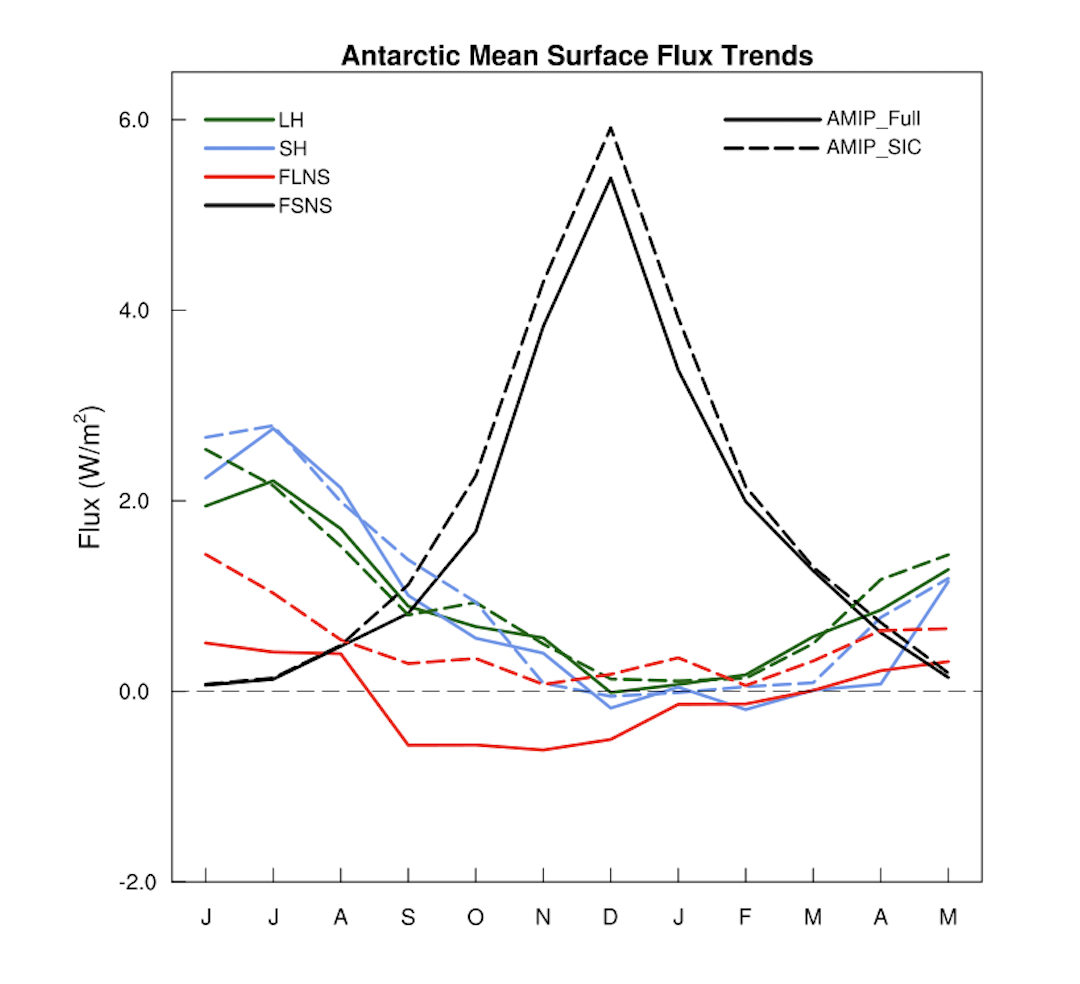


**Fig. S6.** Seasonal changes in the forced Antarctic averaged surface energy budget. Ensemble-mean monthly responses of the surface energy budget terms in the two experiments averaged over the Southern Ocean (all grid boxes south of 60°S with > 50% ocean cover), including the surface latent heat flux (LH), sensible heat flux (SH), net longwave radiation (FLNS), and net short-wave radiation (FSNS) in the AMIP_Full and AMIP_SIC experiments. Fluxes are defined positive in the upward direction.


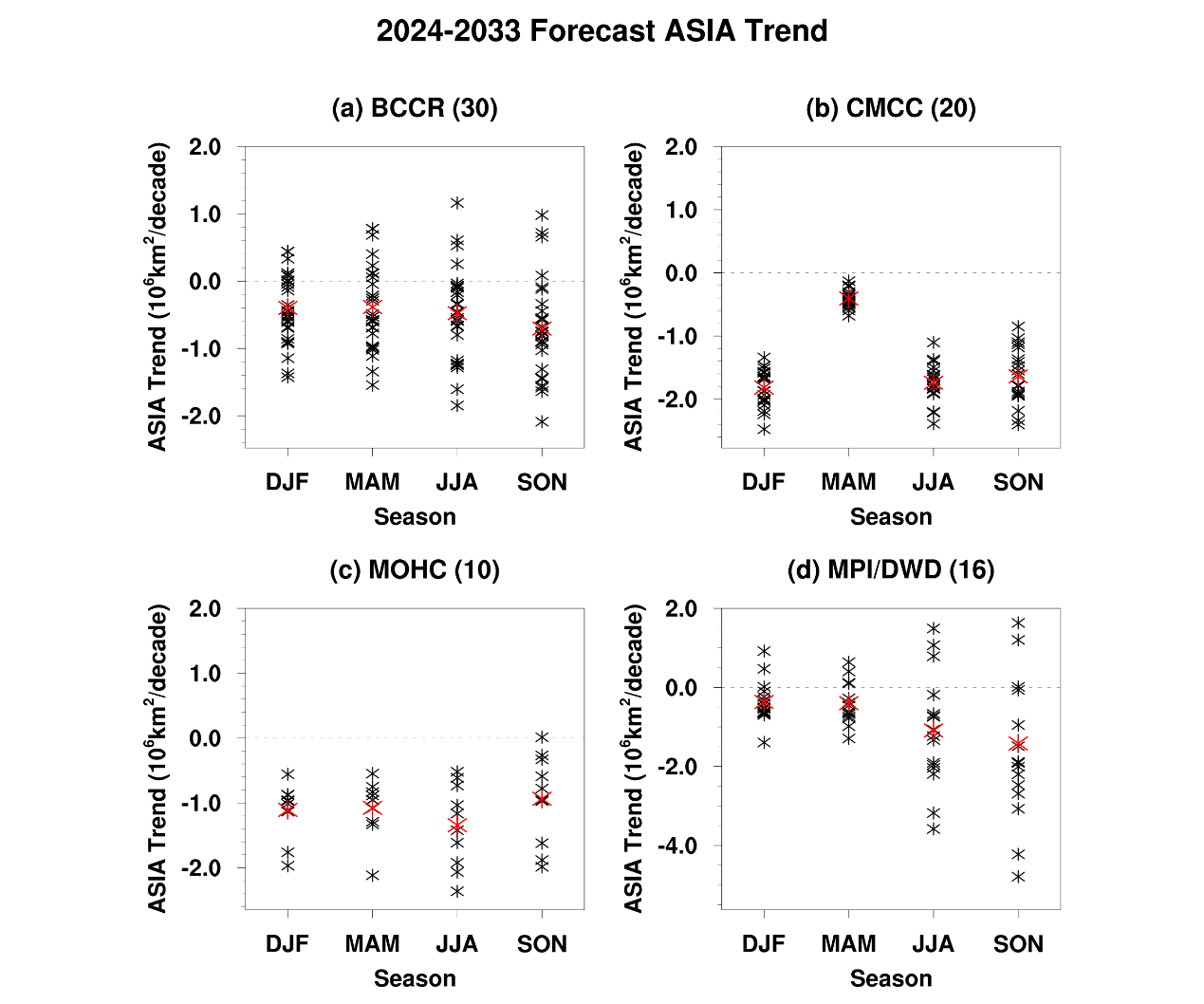


**Fig. S7.** Inter-model and intra-model spread of decadal (December 2024–November 2034) trends in Antarctic sea ice area (ASIA) across four prediction systems: (a) BCCR (30 members), (b) CMCC (20 members), (c) MOHC (10 members), and (d) MPI/DWD (16 members). Black dots denote individual ensemble members, and red crosses indicate the ensemble mean for each season (DJF, MAM, JJA, SON).


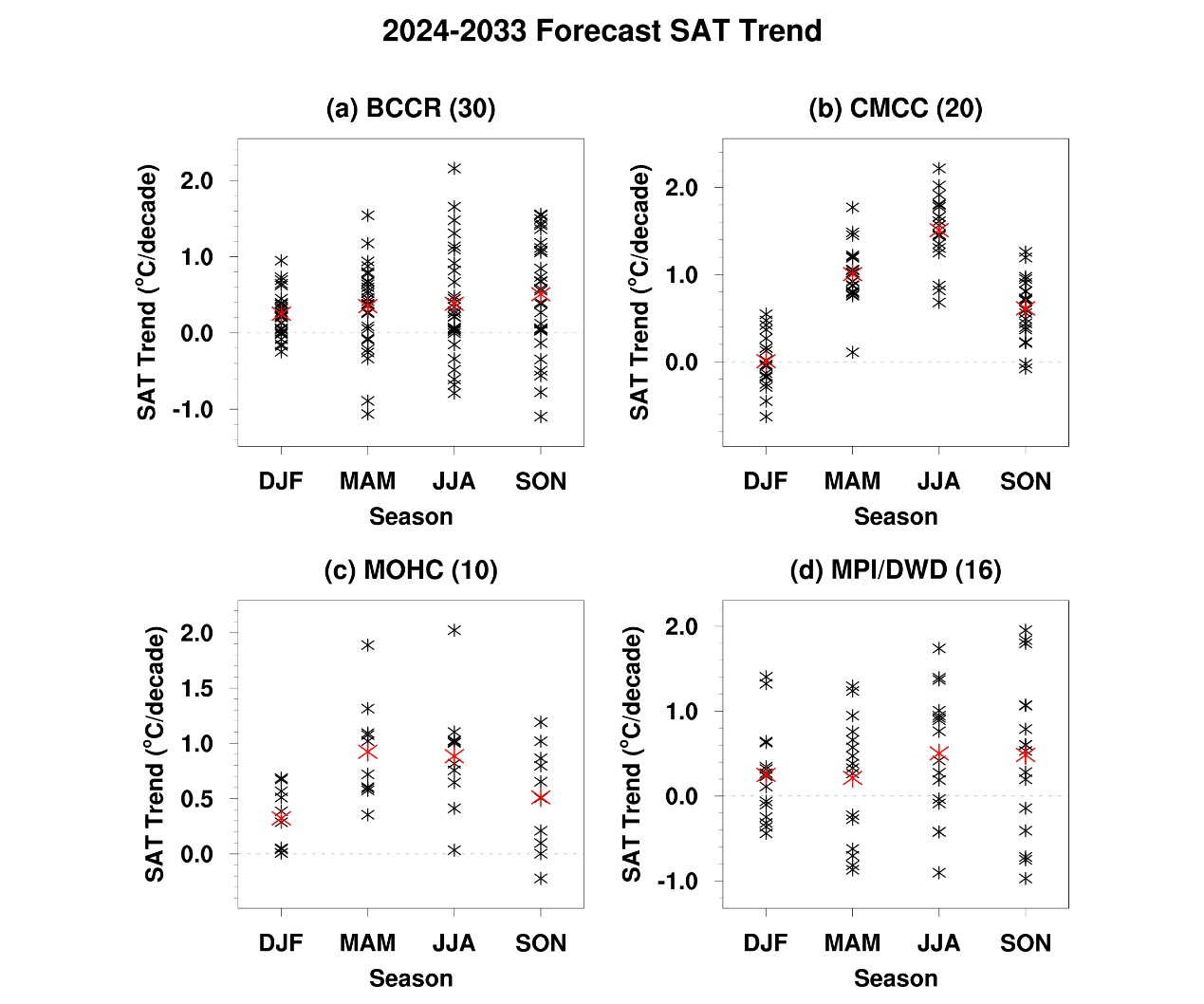


**Fig. S8.** Inter-model and intra-model spread of decadal (December 2024–November 2034) trends in Antarctic surface air temperature (SAT) across four prediction systems: (a) BCCR (30 members), (b) CMCC (20 members), (c) MOHC (10 members), and (d) MPI/DWD (16 members). Black dots denote individual ensemble members, and red crosses indicate the ensemble mean for each season (DJF, MAM, JJA, SON). Trends are in °C/decade.


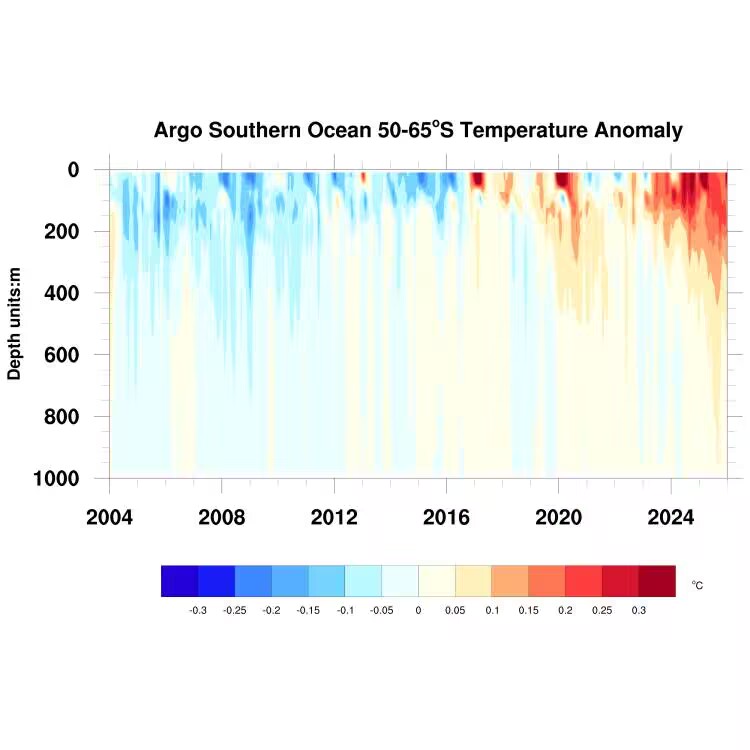


**Fig. S9.** Observed Southern Ocean subsurface temperature anomaly. Seasonal mean time series of subsurface temperature in the Argo float data from January 2004 to December 2025, in degrees Celsius averaged over 50–65°S in the whole Southern Ocean.


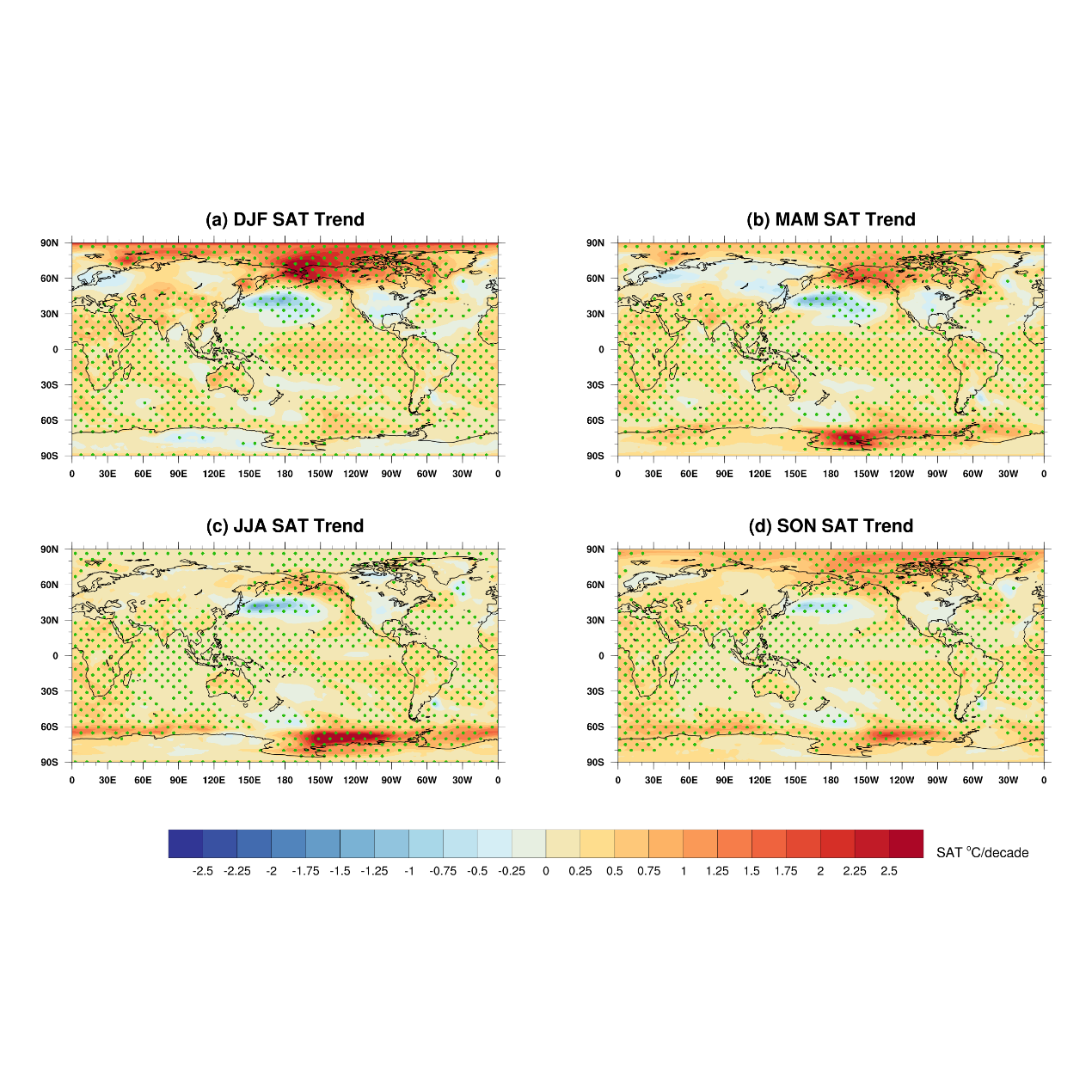


**Fig. S10.** Predicted 10-year linear trends of seasonal mean surface air temperature (SAT) from the decadal forecast ensembles for (a) DJF, (b) MAM, (c) JJA, and (d) SON from December 2024 to November 2034. Stippling indicates regions where the trends are statistically significant at the 5% significance level.


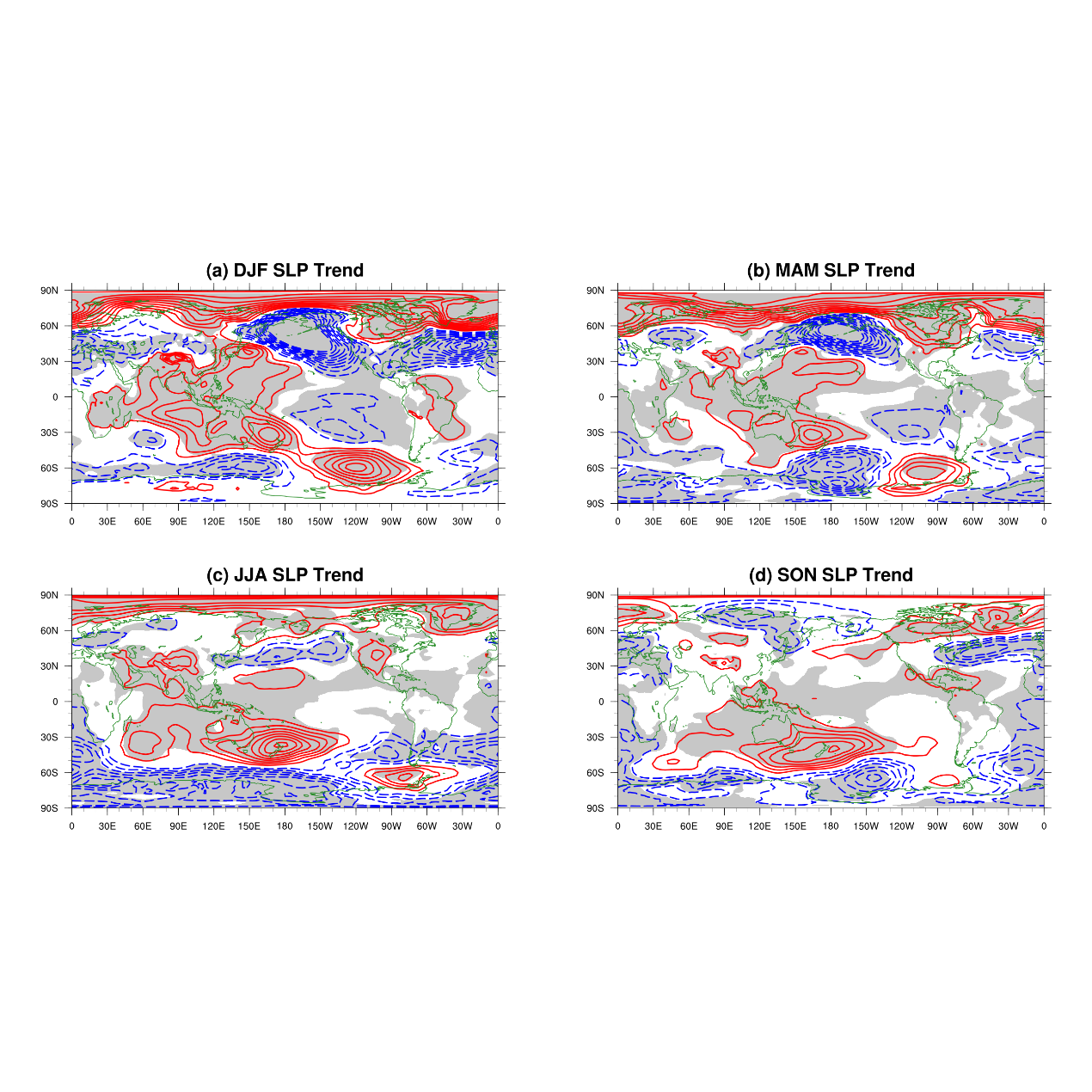
**Fig. S11.** Predicted 10-year linear trends of seasonal mean sea level pressure (SLP) from the decadal forecast ensembles for (a) DJF, (b) MAM, (c) JJA, and (d) SON from December 2024 to November 2034. Contours are drawn at 0.25 hPa/decade intervals, with red solid (blue dashed) lines indicating positive (negative) trends; zero contours are omitted. Shading denotes regions where the trends are statistically significant at the 5% significance level.

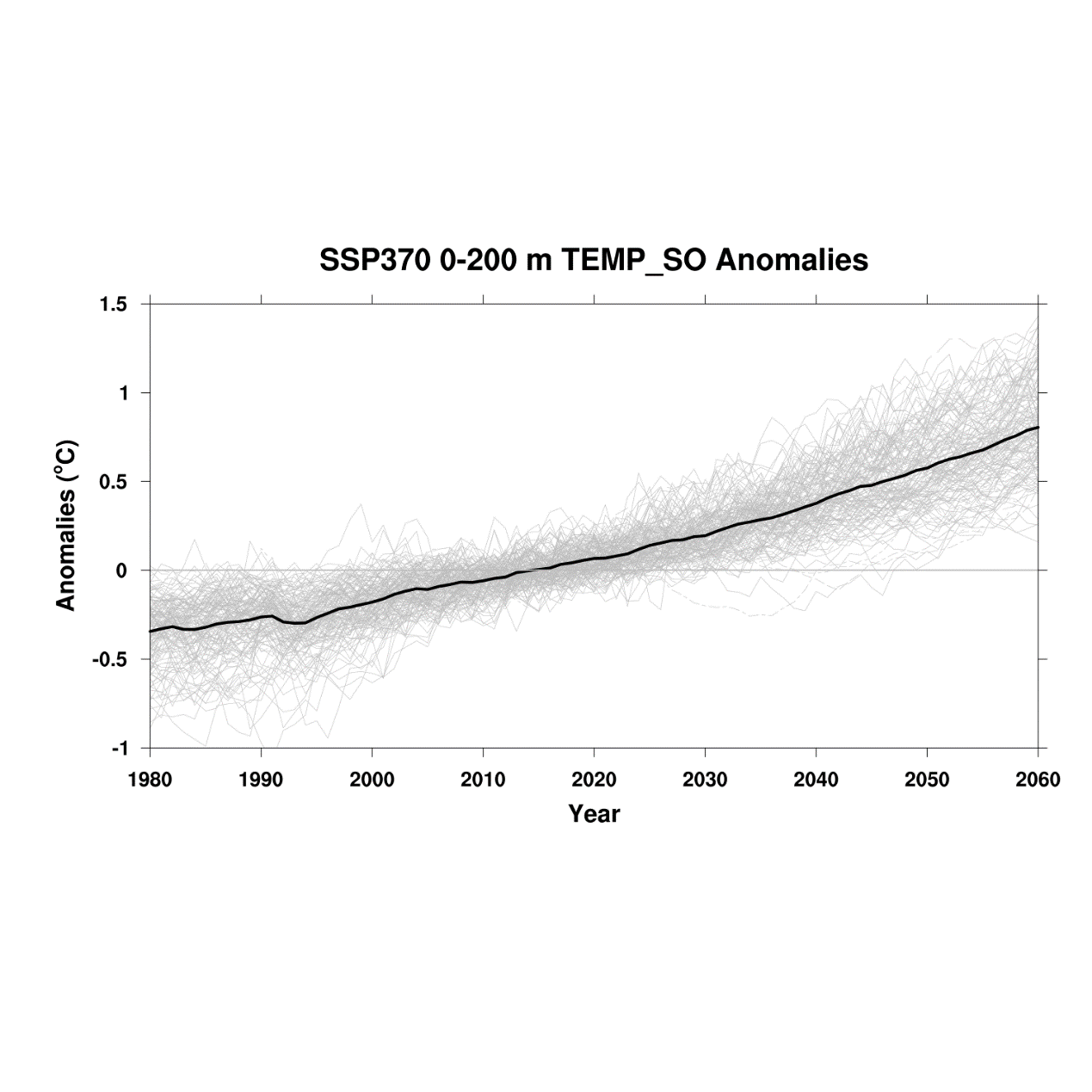


**Fig. S12.** Time series of Southern Ocean temperature anomalies averaged over 50–65°S (TEMP_SO) at the 0–200 m depth, from 1980 to 2060 under the SSP3-7.0 scenario. Grey lines indicate individual ensemble members from CMIP6 simulations, while the black line denotes the ensemble mean. Anomalies are calculated relative to the 2004–2023 climatology. The ensemble mean exhibits a persistent subsurface warming trend, whereas the spread across ensemble members reflects substantial internal variability superimposed on the externally forced signal.


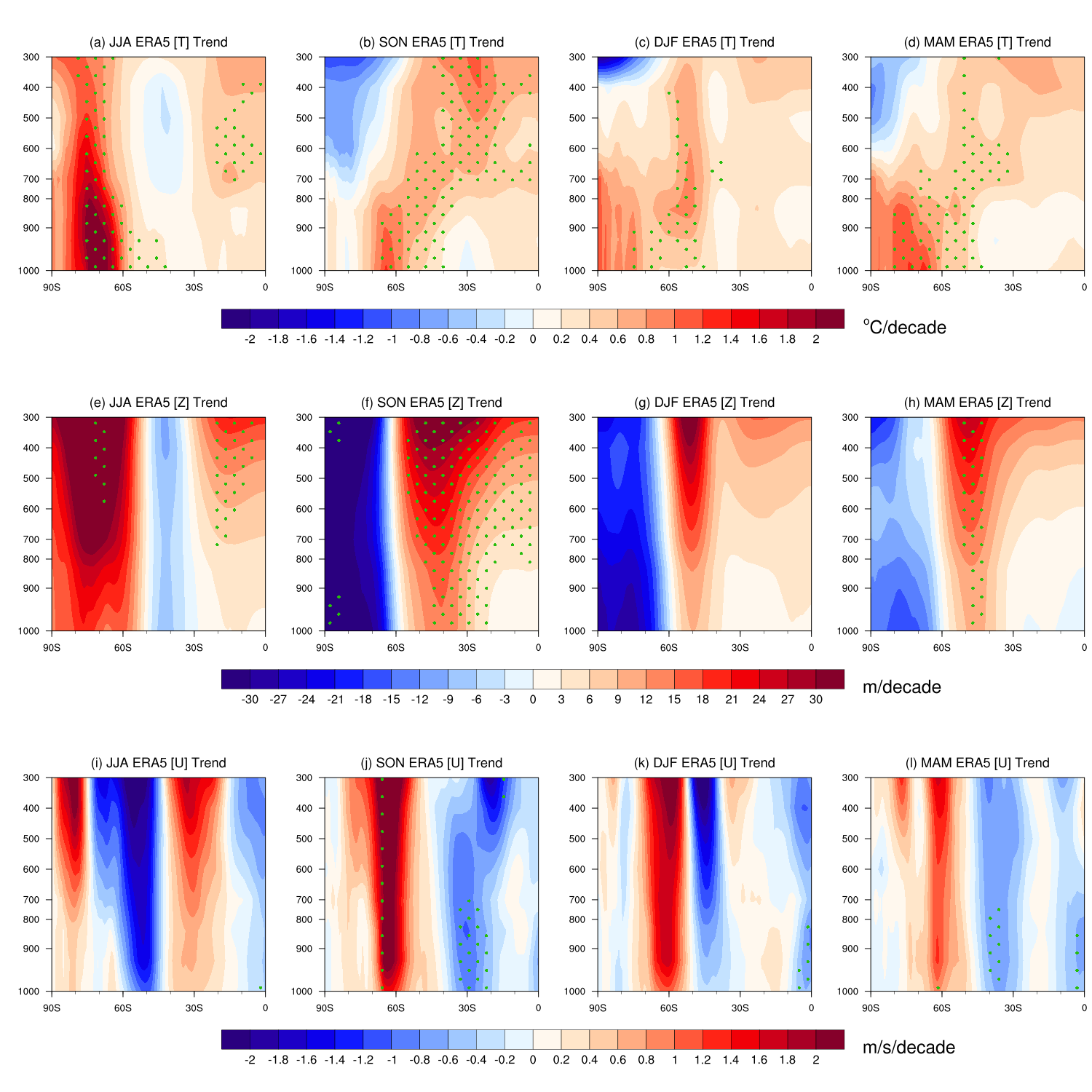


**Fig. S13.** **Observed trends in Southern Hemisphere zonal-mean fields.** 12-year Trends of zonal-mean (a-d) air temperature [T], (e-h) geopotential height [Z], and (i-l) zonal wind [U] in JJA, SON, DJF and MAM in ERA5 from June 2013 to May 2025. Stippling indicates trends significant at the 10% significance level.


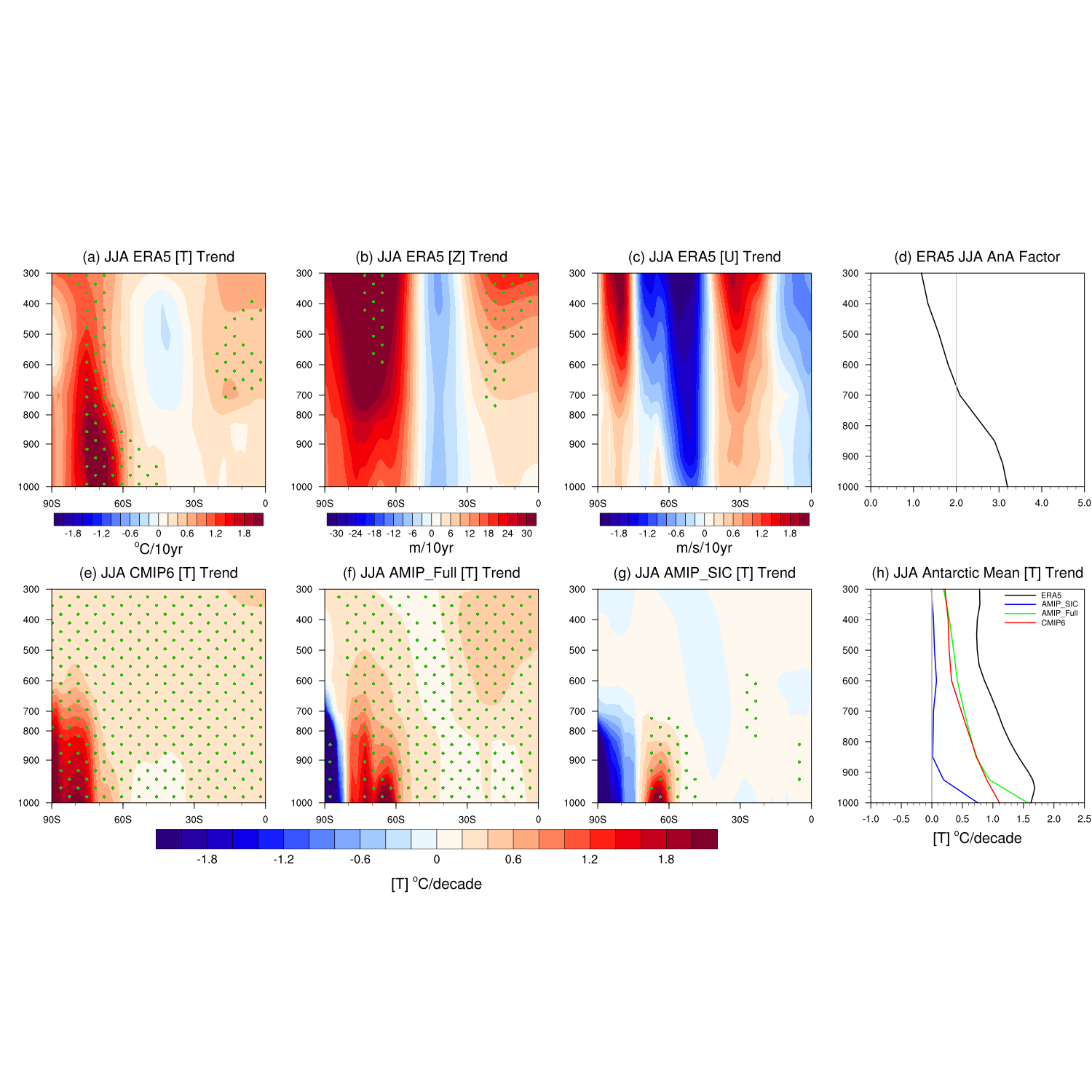


**Fig. S14.** **Observed and forced Antarctic changes in zonal-mean fields in austral winter.** (a-c) 12-year trends in fields of zonal-mean air temperature [T], geopotential height [Z], and zonal wind [U] in JJA from June 2013 to May 2025, (d) Profile of the ratio of the Antarctic mean [T] trend to that of the global mean [T] in each level in JJA, (e-g) Forced trends of JJA [T] in the CMIP6, AMIP_Full, AMIP_SIC simulations; (h) Profile of the Antarctic mean [T] trend in ERA5 and three experiments. Stippling indicates trends significant at the 10% level in (a-c) and 5% level in (e-g).
